# Senescence and programmed cell death in plants: polyamine action mediated by transglutaminase

**DOI:** 10.3389/fpls.2014.00120

**Published:** 2014-04-07

**Authors:** Stefano Del Duca, Donatella Serafini-Fracassini, Giampiero Cai

**Affiliations:** ^1^Department of Biological, Geological and Environmental Sciences (Botany), University of BolognaBologna, Italy; ^2^Department of Life Sciences, University of SienaSiena, Italy

**Keywords:** flower corolla, hypersensitive response, leaf, pollen, polyamines, programmed cell death, senescence, transglutaminase

## Abstract

Research on polyamines (PAs) in plants laps a long way of about 50 years and many roles have been discovered for these aliphatic cations. PAs regulate cell division, differentiation, organogenesis, reproduction, dormancy-break and senescence, homeostatic adjustments in response to external stimuli and stresses. Nevertheless, the molecular mechanisms of their multiple activities are still matter of research. PAs are present in free and bound forms and interact with several important cell molecules; some of these interactions may occur by covalent linkages catalyzed by transglutaminase (TGase), giving rise to “cationization” or cross-links among specific proteins. Senescence and programmed cell death (PCD) can be delayed by PAs; in order to re-interpret some of these effects and to obtain new insights into their molecular mechanisms, their conjugation has been revised here. The TGase-mediated interactions between proteins and PAs are the main target of this review. After an introduction on the characteristics of this enzyme, on its catalysis and role in PCD in animals, the plant senescence and PCD models in which TGase has been studied, are presented: the corolla of naturally senescing or excised flowers, the leaves senescing, either excised or not, the pollen during self-incompatible pollination, the hypersensitive response and the tuber storage parenchyma during dormancy release. In all the models examined, TGase appears to be involved by a similar molecular mechanism as described during apoptosis in animal cells, even though several substrates are different. Its effect is probably related to the type of PCD, but mostly to the substrate to be modified in order to achieve the specific PCD program. As a cross-linker of PAs and proteins, TGase is an important factor involved in multiple, sometimes controversial, roles of PAs during senescence and PCD.

## INTRODUCTION

The functions exerted by polyamines (PAs) in plants have been reviewed along the first years of research by several pioneers in the field (Bagni and Serafini-Fracassini, 1974; Smith et al., 1979; Smith, 1985; Friedman et al., 1986; Galston and Kaur-Sawhney, 1987; Bagni, 1989; Evans and Malmberg, 1989; Egea-Cortines and Mizrahi, 1991; Tiburcio et al., 1993) starting from the first paper dealing with the stimulatory effect of the three aliphatic PAs (putrescine, PU; spermidine, SD; spermine, SM) on tuber dormant tissues (Bertossi et al., 1965). More recently, in addition to many excellent reviews, a special issue dedicated to PAs in plants has been published by several scientists of the field, dealing with transport, metabolism, stress tolerance, growth, senescence, unusual PAs, thermospermine, chemoprevention, and conjugated PAs (Various authors, 2010). The general idea is that growth phenomena are regulated by a cohort of environmental and internal factors among which PAs, essential juvenilation growth substances in all living organisms that regulate differentiation, organogenesis, reproduction and cell proliferation in higher plants and algae, as well as senescence, PCD, and homeostatic adjustments in response to external stimuli and stresses.

**FIGURE 1:**
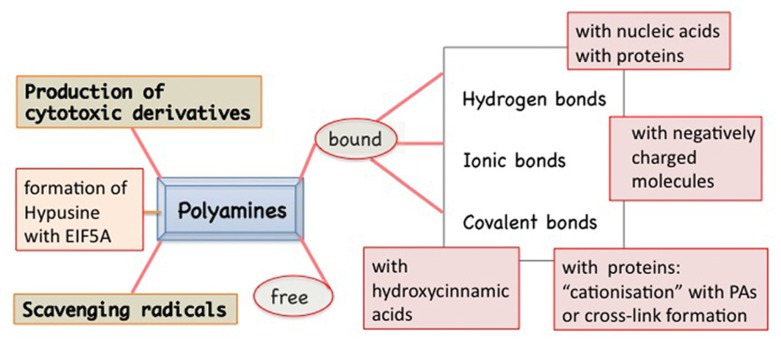
**Polyamines (PAs) in plants exist in two main forms: free and bound to many other molecules by different types of bindings: hydrogen type, more or less polarized, ionic type, or covalent one.** The covalent linkage catalyzed by transglutaminase to specific proteins is Ca^2^^+^ dependent. Peculiar of some families of plants is the PA linkage with hydroxycinnamic acids. PAs are also actively metabolized: from PU to SD and SM but also vice versa. In addition, they can give rise to cytotoxic derivatives. PAs can act as free radicals. SD can be metabolized, by removing the aminopropyl group, and linked to a lysyl residue of the EIF5A precursor thus forming a peculiar derivative, the hypusine.

Polyamines are present in the cells in free and bound form (**Figure 1**). In fact, these polycations are able to form linkages of various types and strength with several molecules. In addition to ionic linkages with negatively charged molecules, interactions may occur by electrostatic linkages, causing conformational stabilization/destabilization of DNA, RNA, chromatin, and proteins. Covalent bonds give rise to the formation of hypusine, insoluble complexes, and “cationization” or formation of cross-links between proteins (e.g., cytoskeleton as in animals), but also with photosynthetic complexes and hydroxy-cinnamic acids, specific of plants. PAs act as free radical scavengers and some of their derivatives might result cytotoxic (**Figure 1**). These multiple aspects of PAs reflect on their roles in the cell life and several of them are related to the senescence progression and PCD both in animals and in plants. Very recently, a review on PAs and PCD in both organisms has been published, dealing prevalently with the consequences of PA oxidation and their cytotoxic products, but ignoring completely the aspect of PA binding (Moschou and Roubelakis-Angelakis, 2013).

## SENESCENCE AND PROGRAMMED CELL DEATH

Programmed cell death (PCD) in plants, like in animals, may be a physiological terminal stage, genetically controlled, of cell differentiation; in some cases the dying cells acquire specific functions (e.g., vascular tissues, fibers) or in contrast cells die after the accomplishment of their role. This kind of PCD could be specifically named developmental cell death (DCD) being related to an internal program of species-specific correct development even though it might also be triggered by adverse environmental factors (Wu et al., 2012). Other types of PCD may occur as the result of external, either biotic or abiotic stimuli, like different environmental signals or pathogen attacks that modify the original cell program. Sometimes, it is difficult to discriminate between programmed and accidental phenomena. In this review, the general term PCD will be used if not otherwise specified. Confusion is also centred on the application of the terms senescence and PCD that may be considered separate, partially overlapping or even identical events (Thomas et al., 2003a; van Doorn and Woltering, 2005; Rogers, 2006). Senescence is not necessarily a stage of PCD; when senescence takes place, it is not a steady state but a gradual evolution of the entire cell and even though sometimes it can be delayed or reversed, usually is preliminary to PCD.

The DCD can involve the co-ordinated death of the cells of an entire vegetative or reproductive organ, or part of it, and might in some cases cause its abscission, preceded by the remobilization of most of the nutrients. DCD can be accompanied by nuclear condensation, membrane blebbing, occasionally DNA fragmentation and cysteine protease activity (Serafini-Fracassini et al., 2002; Ye et al., 2013). At the subcellular level, mitochondria may play a central role, retaining their function during senescence, since respiration continues by alternative oxidase (Vanlerberghe, 2013). Increase of ROS production and protease and nuclease activities have been reported during the leaf senescence (Quirino et al., 2000; Rubinstein, 2000). The cross-talk of nitric oxide and reactive oxygen species in plant PCD (Wang et al., 2013) as well as catabolic and interconversion products of PAs (Moschou and Roubelakis-Angelakis, 2013) have been recently reviewed. In green tissues, chloroplasts swell and re-differentiate into gerontoplasts characterized by the dismantling of thylakoidal membrane; thus, proteins, chlorophylls, lipids as well as nucleic acids are degraded and the photosynthetic activity decreases. Whereas mitochondria catabolize lipids deriving from thylakoids, vacuoles (which represent the lytic compartment of the plant cell) play relevant roles in the autophagic degradative metabolisms, as exemplified for chlorophyll, and finally rupture of tonoplast membrane takes place causing the release of degradative enzymes (van Doorn and Woltering, 2010). Cell walls of some specialized cells, before the cell dies, frequently undergo secondary modifications, such as lignification, suberification, and gelification.

During senescence, the levels of PAs are not constant showing peaks especially at its beginning, but thereafter PAs usually decrease (Galston and Kaur-Sawhney, 1990; Cohen, 1998). This pattern however depends on the type of senescence model, if induced by external factors or natural.

Much data on the PA effect were obtained through the exogenous supply of PAs, PA analogs or from loss of function or mutants in PA metabolism genes. In animal systems, the role of free PAs in apoptosis is still controversial; contrarily to animal cells, plant cells can be less affected by excess PAs, in some plant families by binding them to TCA-soluble conjugates, such as cinnamic acids, or by storing them in the vacuole (Bagni and Tassoni, 2001). In plants, PAs can allow a prolonged survival of excised organs such as leaves, flowers, and fruits even though, as in animals, some contradictory data are reported (Altman and Bachrach, 1981; Galston and Kaur-Sawhney, 1987; Bagni and Pistocchi, 1989; Legocka and Zajchert, 1999; Lester, 2000; Hanzawa et al., 2000; Mehta et al., 2002; Bagni and Tassoni, 2006; Tassoni et al., 2006; Kusano et al., 2008; Muñiz et al., 2008; Nambeesan et al., 2010; Serafini-Fracassini et al., 2010). Examples are the different types of PCD of excised or senescing leaves and protoplasts (Galston and Kaur-Sawhney, 1990; Besford et al., 1993) or aged leaf disks (Legocka and Zajchert, 1999; Serafini-Fracassini et al., 2010), as well as vessels (Muñiz et al., 2008; Vera-Sirera et al., 2010) incompatible pollen/style system (Del Duca et al., 2010; Gentile et al., 2012) and senescing flowers (Serafini-Fracassini et al., 2002; Bagni and Tassoni, 2006; Tassoni et al., 2006; Della Mea et al., 2007a,b). Thermospermine is a structural isomer of spermine first discovered in thermophilic bacteria (Oshima, 1979). Thermospermine is critical for proper vascular development and xylem cell specification, in preventing premature maturation and death of the xylem vessel elements (Vera-Sirera et al., 2010).

The formation of hydrogen peroxide and cytotoxic products via PA catabolism is considered as one possible mechanism of PA involvement in PCD (Yoda et al., 2003, 2006) and the ability of plants to control stress is related to their capacity to metabolize PAs (Alcazar et al., 2010). In addition to the known functions of PAs in PCD by prevention of membrane damage, retard of nucleic acid and protein degradation, including the chloroplast photosystems, or acting as free radical scavengers (here not described, see the above reviews), PAs could exert their roles also by other mechanisms of action. Thus, in order to re-interpret at least some of the effects of PAs in PCD models above reported and to obtain new insights into their molecular mechanisms, their conjugation to proteins has been revised here.

## POST-TRANSLATIONAL MODIFICATION OF PROTEINS: THE TRANSGLUTAMINASE ENZYMES

**FIGURE 2:**
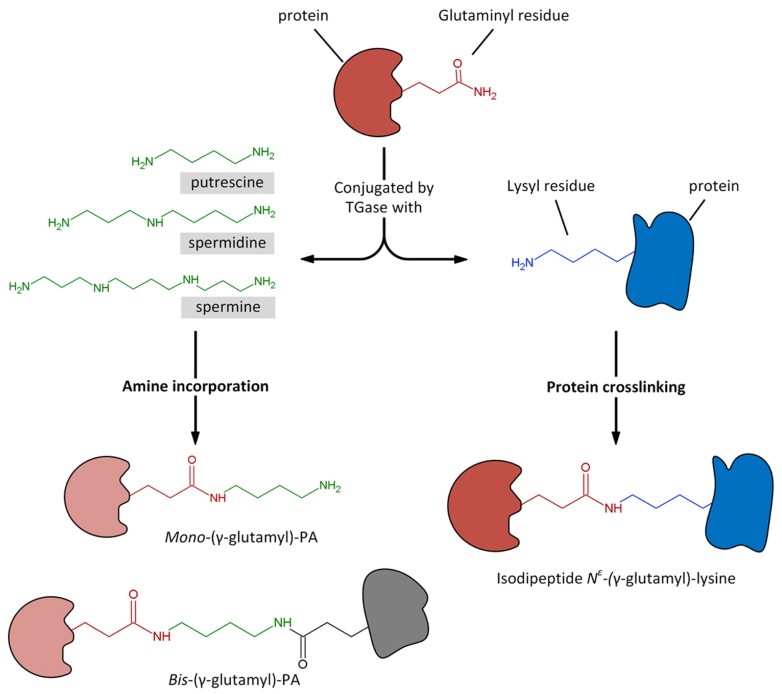
**Transglutaminase could catalyze the Ca^2+^-dependent incorporation of substrates having a primary amino group, as polyamines, to the γ-carboxamide group of a specific protein-bound glutaminyl residue, giving rise to *mono*- and *bis*-(γ-glutamyl)-PAs.** Similarly, the enzyme catalyzes the acyl-transfer reaction between the γ-carboxamide group of a specific protein-bound glutaminyl residue and the ∊-amino group of a distinct protein-bound lysyl residue, giving rise to protein crosslinking by the formation of isodipeptide N^∊^-(γ -glutamyl)-lysine.

**FIGURE 3:**
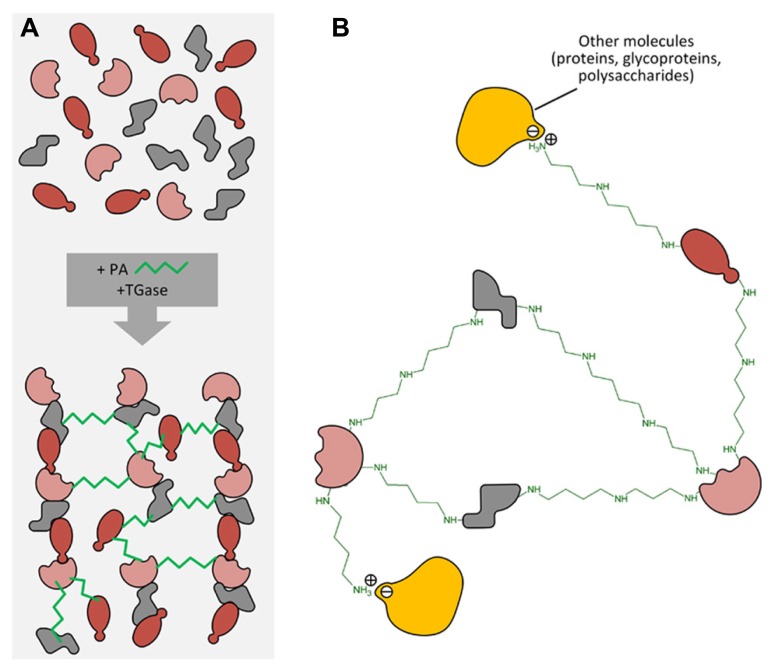
**(A)** Different protein molecules can form a net by PA linkage catalyzed by transglutaminase. **(B)** PAs can also form other linkages (i.e., ionic ones) with different molecules such as proteins, polysaccharides, phenols, etc., thus forming heterogeneous complexes.

It is hypothesized that PAs exert some of the above-described effects through a biochemical process of conjugation with proteins. This activity is catalyzed by the enzyme transglutaminase (TGase). The process of transamidation is part of a set of post-translational modifications to which proteins can be subjected and include a number of efficient regulation strategies, such as phosphorylation/dephosphorylation, covalent modification, proteolytic degradation or activation, interaction with partner proteins. At recent, the post-translational modification is identified as one of the most important, rapid, and precise methods by which eukaryotic cells respond to environmental stresses or developmental changes. The covalent linkages of PAs to proteins are catalyzed by the enzyme family of TGase (R-glutaminylpeptide-amine γ-glutamyltransferase; E.C. 2.3.2.13), discovered and studied in animals since many years (Sarkar et al., 1957; Folk, 1980; Lorand et al., 1988). TGases are present in eukaryotic and prokaryotic organisms; in animals they fulfil different enzymic functions as summarized in a book edited by Mehta and Eckert (2005) and also reviewed (Griffin et al., 2002; Lorand and Graham, 2003; Beninati et al., 2009). Transglutaminase 2 (TG2) is the most widely distributed member of the transglutaminase family with almost all cell types in the body; TG2 is an extremely versatile protein exhibiting transamidating, protein disulfide isomerase and guanine, and adenine nucleotide binding and hydrolyzing activities. TG2 can also act as a protein scaffold or linker. This unique protein also undergoes extreme conformational changes and exhibits localization diversity (Gundemir et al., 2012). One of the TGase activities, the transamidation catalysis, consisting in the covalent conjugation of PAs and other amine-donors (among which lysyl-residues) to γ-carboxamide groups of protein endo-glutamine residues (Folk, 1980; Beninati and Folk, 1988), is the activity that has been most extensively studied in plants (reviewed by Del Duca and Serafini-Fracassini, 2005; Serafini-Fracassini and Del Duca, 2008; Del Duca et al., 2014). PU, SD, and SM differ in both their number of positive charges exhibited at the cell physiological pH (2 in PU, 3 in SD, and 4 in SM) and their backbone length (PU: 6.5 Å; SD: 11.12 Å; SM: 14.6 Å). Their two terminal amino groups conjugate to one or two glutamyl residues giving rise to PA derivatives, either *mono*-(γ-glutamyl)-PAs *(mono*-PAs) or *bis*-(γ-glutamyl)-PAs (*bis*-PAs; **Figure 2**). The additional positive charges introduced by protein-bound PAs due to their internal iminic- or free terminal aminic group (*mono*-PAs) may induce protein conformational changes. *Bis*-PA derivatives can form both inter- and intra-molecular cross-links in proteins. The backbone length of the PAs determines the length of the cross-link it forms: *bis*-(γ-glutamyl)-SD (*bis*-SD) bridges, and even more so those involving *bis*-(γ-glutamyl)-SM (*bis*-SM), span greater distances than those formed by *bis*-(γ-glutamyl)-PU (*bis*-PU). The link formed between glutamyl and lysyl residues is much shorter that those involving PAs (**Figure 2**). The binding is highly specific and is probably primarily dependent on the substrate conformation (Griffin et al., 2002). *Mono*-PA production is affected by PA concentration since high levels of PAs saturate the acyl donor residues of the substrate proteins limiting the formation of *bis*-derivatives. In this sense, the levels of PAs have a critical role in the modulation of the number of protein cross-links formed. Since several PA molecules can cross-link more proteins simultaneously, high molecular complexes may form (**Figure 3**). In addition, the free terminal amino group of *mono*-PAs can interact by additional linkages, for example with negatively charged groups of other types of molecules, thus forming heterogeneous complexes (**Figure 3**). The supramolecular nets of linked proteins are resistant to mechanical stress and proteolysis (Martinet et al., 1990) and are observed frequently as product of TGase cross-linking activity.

## THE TRANSGLUTAMINASES IN PLANTS

After the discovery of PAs conjugated to proteins in plants about 30 years ago (Serafini-Fracassini and Mossetti, 1985; Mossetti et al., 1987), TGase-like activities were detected to catalyze PA conjugation to proteins (Icekson and Apelbaum, 1987; Serafini-Fracassini et al., 1988). In particular, Mizrahi et al. (1989) observed that PAs delayed senescence in oat and *Petunia* leaves and found PAs strongly bound to proteins of high molecular weight. Further on, the identification of the TGase typical products (namely the PA-derivatives), the stimulation by Ca^2^^+^ and the inhibition by EGTA or EDTA, the immunorecognition by TGase antibodies of animal origin, the inhibition by specific inhibitors, and DTT dependence confirmed the identification of this enzyme (Del Duca et al., 1994, 1995; Lilley et al., 1998; Della Mea et al., 2004a). A family of TGases of different molecular mass are located in various organs of higher plants, such as seeds, pollens, meristems, mature vegetative organs, flowers, and petals. The enzymes are very active in chloroplasts where react to external stimuli, among which light, but they are active also in the cytoplasm, in relationship with cytoskeleton, in cell wall and probably in mitochondria. These topics have been reviewed by Del Duca and Serafini-Fracassini (2005), Serafini-Fracassini and Del Duca (2008), Del Duca et al. (2013b, 2014).

No DNA sequence sharing homology with the well-known animal TGases had been found in the databases of several plants making difficult identifying plant TGases by sequence comparison. A computational analysis has shown the presence in *Arabidopsis thaliana* of only one gene, *AtPng1p*, that contains the TGases catalytic domain with the Cys–His–Asp triad. This gene encodes a putative N-glycanase, active at least *in vitro* in heterologous systems (Diepold et al., 2007; Masahara-Negishi et al., 2012) but its product also acts as a TGase, having a Ca^2^^+^- and GTP-dependent transamidase activity and forming glutamyl-PA derivatives (Della Mea et al., 2004b). This was the first plant protein, isolated and characterized at the molecular level, displaying a TGase activity, whose biochemical parameters and 3D structure agree with those typically exhibited by animal TGases. Other TGases of different molecular mass of chloroplasts origin were sequenced (Villalobos et al., 2004; Campos et al., 2013) and the homology of the amino acidic composition of three TGases of *Helianthus tuberosus* meristems with mammal TGases were reported (Beninati et al., 2013).

This review is devoted to the possible role of plant TGase and, consequently, of conjugated PAs, in senescence and PCD. Thus, for other aspects of TGase in plants not strictly related with these subjects, other reviews can provide more information (Del Duca and Serafini-Fracassini, 2005; Serafini-Fracassini and Del Duca, 2008; Della Mea et al., 2009; Serafini-Fracassini et al., 2009; Del Duca et al., 2013b, 2014). One of the possibilities that thermospermine regulates xylem terminal differentiation through the interaction with yet unidentified elements, as suggested by Vera-Sirera et al. (2010) and that also thermospermine is conjugated to proteins by TGase, is a promising hypothesis that cannot be discussed as unfortunately no experimental data are available. Due to the relatively recent and still incomplete data on plant TGases in PCD, references to this subject in animals will be given for comparison and for suggesting possible interpretations.

## TRANSGLUTAMINASE IN PROGRAMMED CELL DEATH

One of the most studied mammalian TGase, tissue TGase (tTGase or TG2) is constitutively expressed but has a low activity in growing animal cells and/or in non-stressed cells, whereas it is generally up-regulated in cells undergoing cell death. As an example, TG2 is involved in the formation of apoptotic bodies in which *N*^∊^(γ-glutamyl)lysine cross-links have been detected. However, due to its multifunctional activity as TGase but also as Ca^2^^+^-independent GTPase, it also acts as effector in the prevention of cell death. Over-expression of TG2 in cells leads to increased export of the enzyme to the cell surface and into the extracellular matrix (Griffin and Verderio, 2000; Wang et al., 2012). Activation of the enzyme by increase of cytosolic Ca^2^^+^ would result in the cross-linking of both intracellular and extracellular proteins leading to stabilization of the dying cell and surrounding matrix thus maintaining both cellular and tissue integrity or remodeling. Expression of the enzyme has been generally correlated to both the level of nuclear fragmentation and to morphological changes of cells undergoing apoptosis. The extensive polymerization into insoluble aggregates of actin, retinoblastoma gene product, and nuclear proteins is a key signal for the initiation of apoptosis (Oliverio et al., 1997). This could be important for preventing the inflammatory responses that would follow the dispersion of the contents of dying cells. However, the evidence that TG2 is likely implicated as a mediator of apoptosis is conflicting (Fesus and Szondy, 2005). It was proposed that the pro-apoptotic or anti-apoptotic effect of TG2 is dependent on the activation pathways and location; nuclear and extracellular TG2 may affect anti-apoptosis while cytosolic TG2 is pro-apoptosis (Milakovic et al., 2004). Intracellular SM and SD are capable of modulation of TG2 expression (Chen and Mehta, 1999). However, the blockage of PA synthesis in different cell types was shown to differently influence TG2 expression by decreasing expression in one cell type and increasing expression in another (McCormack et al., 1994; Wang et al., 1998; Piacentini et al., 2005). It is becoming evident that the multifunctional roles of TG2, both cytosolic and nuclear TG2, in cell death processes (apoptosis and or autophagy) are dependent upon the cell type, stimuli, subcellular localization, and conformational state of the protein. The conformational and functional diversity of TG2 in the context of its role in numerous cellular processes has been recently reviewed by Gundemir et al. (2012); in particular, it has been highlighted how differential localization, conformation and activities of TG2 may distinctly mediate cell death processes.

In plants, an increasing number of reports on TGase in senescence and PCD, studying both reproductive and vegetative organs, suggest a strict correlation of TGase and PA physiological effects. Until now, studies have been mainly focused on senescence and death of the leaf (Sobieszczuk-Nowicka et al., 2007, 2009; Serafini-Fracassini et al., 2010; Sobieszczuk-Nowicka and Legocka, 2014) and of the flower petals (Serafini-Fracassini et al., 2002; Della Mea et al., 2007a,b) or on the growing or abiotic stress-induced dyeing pollen (Iorio et al., 2012b) as well as self-incompatible (SI) pollination system (Del Duca et al., 2010; Gentile et al., 2012) and on the hypersensitive response (IR) to pathogens (Del Duca et al., 2007). The study of these plant models is important not only for basic but also for applied research. Just as an example, freshly cut leaves, which are utilized as food, like lettuce, and freshly cut ornamental flowers have in fact a short shelf life.

### THE FLOWER COROLLA PCD

In reproductive organs, various parts undergo DCD. Petals, which are modified leaves, have in general a vexillary role and, once completed this role, they enter senescence and fall; in some cases, e.g., *Nicotiana*, they remain *in situ*, become rigid and papyraceous to protect the initial growth of the ovary. Flower petal senescence and its final death is a highly regulated developmental phase, controlled by hormones and growth factors, like ethylene, cytokinins, abscisic acid (Orzaez et al., 1999; van Doorn and Woltering, 2008; Amasino and Michaels, 2010; Rogers, 2006, 2012) as well as PAs, as above reported. In the long-lived flowers, pollination acts as a signal for senescence, while in the short-lived flowers this event is independent from pollination. Petals are histologically rather homogenous consisting of parenchyma, thin veins and a protecting layer of epidermis.

**FIGURE 4:**
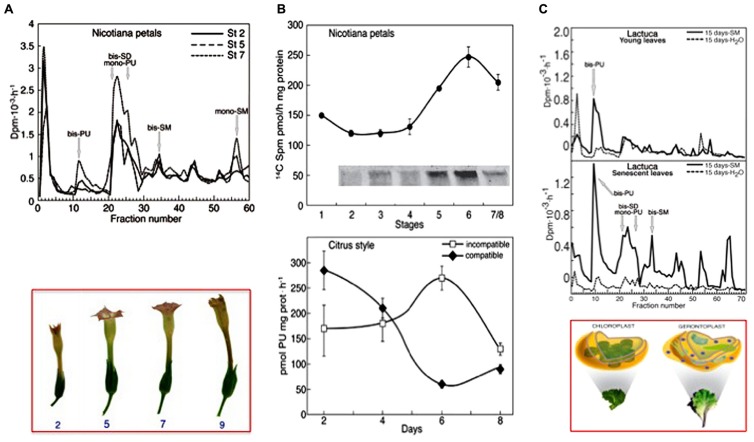
**Transglutaminase activity in different senescing or PCD models. (A)** Corolla petals of flowers of *Nicotiana tabacum* senescing on the plant, starting from stage 2 (growing) to 7 (senescent), bottom panel. Top panel: glutamyl-PAs were separated by ion-exchange chromatography of extracts of the whole corolla at stages 2, 5, and 7 incubated with [^3^H] SM as described in **(B)** [modified from Della Mea et al. (2007b)]. **(B)** Comparison of the TGase pattern in two models of PCD. Top panel: petals of *Nicotiana tabacum* during natural corolla life span (stages 1–8). The activity was detected by incubating corolla extracts in the presence of 0.2 mM [^14^C] SM as tracer and the labelled conjugates produced were either measured by TCA precipitation or, after separation by SDS-PAGE, detected by autoradiography; a detail of the 250 kDa immunorecognized TGase polymers is shown in the insert [modified from Della Mea et al. (2007b)]. Bottom panel: TGase activity in compatible and self-incompatible pollinated styles of *Citrus grandis* at different days of pollination. The activity was detected by incubating style extracts in the presence of [^3^H]-PU as the tracer (modified from Gentile et al., 2012). At day 6, the incompatible pollen tube growth was blocked. **(C)** Senescing leaves of *Lactuca sativa* compared with the young ones. Glutamyl-PAs were separated by ion-exchange chromatography from extracts of young and senescing leaves, sprayed with SM or water when still on the plant and extracted 15 days after the spray. Extracts were incubated for 60 min with [^3^H] SM as tracer in the assay mixture containing 5 mM Ca^2^^+^. Bottom panel: picture of the *Lactuca* leaves and related plastids [modified from Serafini-Fracassini et al. (2010)].

As a flower corolla model, senescence and death were studied *in planta* or in flowers of *Nicotiana tabacum* excised at different growing stages (Serafini-Fracassini et al., 2002; Della Mea et al., 2007a,b). The senescence of corolla follows a visible acropetal gradient, completed by the death of the entire corolla that concludes with the teeth curling. The stages of maturation, senescence and death were established macroscopically by analysing various morphological parameters. The timing and localization of the most characteristic events were evaluated by biochemical and physiological analyses as well as by cytological observations. Even though precocious signs can be detectable also before, flower petal senescence was characterized by the appearance at its base of a “ring”, named abscission zone (AZ), of dying cells, which detach from each other and blocks the sap transport. This event is concomitant with nuclear blebbing, DNA laddering, cell wall modification, peak of protease activity, decline of protein, water and pigment (anthocyanins, chlorophylls) content, decrease in membrane integrity and increase in Ca^+^^+^-dependent TGase activity, detected as amount of the protein modified by labeled SM and increase in glutamyl-PA (especially *mono*-PU) production (**Figures 4A,B**). *Bis*-PA derivatives can form both inter- and intra-molecular cross-links in proteins, whereas *mono*-PAs are preferential substrates of PA oxidases. The maximum of TGase activity coincides with the appearance of high polymers immunorecognized by TGase antibody and with the flower senescence (**Figure 4B**, insert). These data could support the hypothesis that the formation of more cross-linkages among proteins possibly increased the dimension and strength of the protein net. This could be relevant for structural substrates, like cytoskeleton, discussed below, or cell walls. Ca^2^^+^ could exert an important regulatory role of the enzyme activity. It is known that in senescing tissues this cation increases in concentration (Huang et al., 1997; Ma and Berkowitz, 2011), especially because of its release from the vacuole, caused by tonoplast rupture.

The acropetal gradient of tobacco corolla senescence and DCD was temporally preceded by a maximum of TGase activity, which shifted from the proximal to the distal part of the corolla (Della Mea et al., 2007b). This activity modified either the endogenous substrates alone or a specific recombinant mammal TGase exogenous substrate, namely His6-X Press-green fluorescent protein (GFP); the modifications are revealed by changes in their electrophoretic migration, thus of their molecular mass, and the PA glutamyl derivatives produced. The recombinant GFP is a good substrate for TGase, because its electrophoretic shift changes in a similar way after modification by animal and plant TGases.

The TGase protein bands were immunorecognized by three antibodies raised against mammalian, nematode and *Arabidopsis thaliana* TGases. The fact that the antibody raised against *Arabidopsis* TGase recognizes some proteins of *Nicotiana*, also immunodetected by two animal antibodies, and that plant and animal TGases present similar molecular weights and modify GFP in a similar way, would suggest a similarity among these enzymes. However, plant and animal PCD are dissimilar due to the cell structure; in fact, typical plant organelles, such as chloroplasts, vacuoles, and also possibly the cell walls, play a role in the induction or execution of PCD, as reported during the leaf and petal senescence (Quirino et al., 2000; Rubinstein, 2000; Lim et al., 2007). The localization of TGase in the *Nicotiana* petal cells could suggest new and different roles of this enzyme in PCD in addition to those detected in animal cells. As reported by Della Mea et al. (2007b), a 58-kDa band immunodetected by anti-TGase antibodies, representing also the prevalent form in leaves, decreased during corolla life and was present in the soluble, microsomal, plastidial and cell wall fractions. In contrast, the peak location of a 38-kDa band, mainly a plastidial form, moved progressively from basal to distal parts of the corolla, where it was exclusively present. This 38-kDa putative enzyme could match with a TGase isolated from maize (*Zea mays*) thylakoids (Della Mea et al., 2004a) and with a 39-kDa enzyme detected in chloroplasts of *Medicago sativa* (Kuehn et al., 1991). The plastid TGase, stabilizing the photosystems, could favor the efficiency of photosynthesis and indeed sustain the energy requirements of senescence progression. In the soluble fraction a 52-kDa immuno-positive band was decreasing with age and in late senescence a high (>250 kDa) molecular mass band appeared (Della Mea et al., 2007b), suggesting a binding of the enzyme with a cytoplasmic component (possibly cytoskeletal proteins?) or possibly because of enzyme polymerization as suggested in animals (Lorand and Graham, 2003).

TGase activities were detected in different cell compartments (Della Mea et al., 2007b); activity prevailed in the microsome fraction, where it is in general higher in the proximal part of petals, peaking at the corolla opening, and in the plastids, where it shows an increasing trend. Data on the TGase role in chloroplasts are discussed below in the leaf PCD paragraph. In particular stages of senescence, a minor rate of activity was detected also in the cell walls, prevailing in the distal part and progressively increasing as well as in the soluble fraction, where it is present only in the proximal part at senescence. The intracellular TGase, possibly released into the cell wall as in pollen (see below), was hypothesized to co-operate with cell wall strengthening or modification by protein cross-linking, especially either at the basal abscission zone or distally where the teeth curl, outward during differentiation and then refold at the later stages to protect the developing ovary. During these morphological events, cytoskeleton and turgor changes play a major role, but these are presumably supported by cell wall local strengthening. The walls of the corolla parenchyma cells during senescence undergo modifications evidenced by an increased auto-fluorescence, indicative of its suberification/lignificaton (Serafini-Fracassini et al., 2002) and by the rigid/papyraceous-like aspect of the corolla. Relevant cell wall modifications occur also in cells located in the AZ to prevent the release of toxic substances, desiccation, and pathogen attack after corolla abscission; in fact, the tissues around the AZ must be protected by impermeabilization of the scar. Cell wall could be assimilated to extracellular matrix of senescing animal cells, where TGase stabilize the dying cell and surrounding matrix thus maintaining both cellular and tissue integrity or remodeling. Some data on the TGase in plant cell walls are discussed below and revised by Del Duca et al. (2014). Overall, these data suggest a relationship between DCD and TGase, whose roles are probably different, depending on the function and modification of the compartments in which the enzyme is located.

In PCD, mitochondria have a central role and PAs were long ago detected in these organelles (Torrigiani and Serafini-Fracassini, 1980); PAs were shown to have a role also in *Nicotiana* mitochondria function, as suggested for example by the relationship between SM and mitochondria dysfunction involving the SM-signal pathway (Takahashi et al., 2003). Unfortunately, there are no data on TGase in petal mitochondria; the only data available of a covalent binding of PAs to proteins, tentatively via TGase, in plant mitochondria was obtained in potatoes and mung beans (Votyakova et al., 1999).

In order to evaluate the anti-senescence effects of PAs, detached *Nicotiana* flowers were treated with exogenous SM and with an inhibitor of ethylene action, silver thiosulfate; they showed senescence delay, retard DNA fragmentation and vacuole damage, prolong chloroplast viability with visible preservation of chlorophyll content (Serafini-Fracassini et al., 2002). SM taken up was also converted back to PU and SD, found either in free or TCA-soluble form. In *Nicotiana* these conjugates are mainly hydroxycinnamoyl derivatives, which are known to increase during flowering (Martin-Tanguy et al., 1996), but no evidence is reported of their involvement in senescence. The anti-DCD effect could be mediated, at least in part, by SM covalent binding to TGase substrates. PA supply causes the formation of very high molecular mass products, especially in the presence of an excess of PAs, which cannot be separated by electrophoresis, in addition to different protein bands of lower mass. In animals, many protein substrates were detected among which actin, β -tubulin, annexin, fibronectin and core and H1 histones and others, which could easily be involved in PCD (Piacentini et al., 2005). These proteins could also be substrates of *Nicotiana* TGase; currently, those identified in plants are actin and tubulin, and photosystem ones, like LHCII, as well as some cell wall proteins (Serafini-Fracassini and Del Duca, 2008; Del Duca et al., 2014); thus these substrates are located in different cell compartments, exactly like TGase.

### THE LEAF SENESCENCE AND PCD

Yellowing of leaves is a visible paradigm of leaf senescence and PCD; therefore, leaf is one of the first and more studied models (Quirino et al., 2000; Lim et al., 2007). Once leaves have completed their role, they generally undergo senescence that results in the coordinated degradation of macromolecules and the subsequent mobilization of components to other parts of the plants. Yellowing is well known to be due to the preferential degradation of chlorophyll over carotenoids. Chloroplasts play a role in leaf senescence; they are involved in sustaining the energy requirements for the progression of senescence and develop into gerontoplasts (**Figure 4C**). Concomitantly with chlorophyll release and degradation, ribulose-1,5-bisphosphate carboxylase/oxygenase (RuBisCO) and the light-harvesting-proteins (LHC-P) are also degraded (Park et al., 2007).

Polyamines are known to be also localized in chloroplasts (Bagni and Serafini-Fracassini, 1974) and are therein both synthesized and oxidized (Torrigiani et al., 1986; Bernet et al., 1999). Correlations among levels of PAs in chloroplasts, where their biosynthesis is controlled by white light, chlorophyll biosynthesis and photosynthetic rate have been observed. PAs are involved in the stabilization of thylakoids, in which organic cations are more efficient than Mg^2^^+^ in promoting the stacking adjacent thylakoids (Besford et al., 1993; Legocka and Zajchert, 1999; Ioannidis et al., 2009). PAs have been found associated with PSII, particularly with the LHC and operate on the structure and function of the photosynthetic apparatus during photoadaptation and photoprotection against factors such as UV-B, ozone, etc. (Navakoudis et al., 2007). Thus, their binding seems to be relatively strong and related to precise physiological roles. In *Pisum sativum*, it was found that SM stabilized the molecular composition of the membranes by preventing lipid peroxidation (Stoynova et al., 1999).

The first effects observed of exogenous PA application or PA overexpression on leaves were obtained under stress conditions that may cause PCD. Leaves of monocots and dicots under the influence of osmotic stress lose chlorophyll, undergo rapid senescence, and accumulate large amounts of PU (Galston and Kaur-Sawhney, 1990; Cohen, 1998). The exogenous addition of SD or SM inhibited protein degradation, chlorophyll loss and stabilized thylakoid proteins such as D1, D2, cyt f and the large subunit of RuBisCO (Mizrahi et al., 1989; Besford et al., 1993; Duan et al., 2006). Mizrahi and coworkers also found PAs strongly bound to proteins of molecular weight higher than 45 kDa. Their data suggested that binding could be covalent and stimulated the research on TGase in chloroplasts. An indication of the presence of this enzyme in chloroplasts was at first suggested by Cohen et al. (1982) and detected in leaves by Signorini et al. (1991). Later on in isolated chloroplasts of *Helianthus tuberosus*, TGases have been reported to catalyze the conjugation of PAs to both stromal and thylakoid proteins (LHCII, CP29, CP26, CP24, RuBisCO; Del Duca et al., 1994). The LHCII apoproteins are the preferred substrates, being the oligomeric forms of LHCII much more intensely labeled by PAs than monomeric ones and demonstrating that PAs take part in oligomer stabilization through the formation of cross-links (Dondini et al., 2003). The first plastidial TGase has been identified as a 58-kDa form associated with thylakoids (Del Duca et al., 1994; Dondini et al., 2003); in addition, a 39-kDa Ca^2^^+^-dependent TGase was found to co-purify with LHCII in mature *Zea mays* chloroplasts (Della Mea et al., 2004a). In mature chloroplasts, TGase is activated by light [as demonstrated by the identification of glutamyl derivatives (Del Duca et al., 1995)], salt stress and kinetin (Margosiak et al., 1990; Del Duca et al., 1994; Dondini et al., 2003; Della Mea et al., 2004b; Sobieszczuk-Nowicka et al., 2007). Separation of thylakoid proteins followed by LC-MS identification of protein complexes, confirmed that *Z. mays* chloroplast TGase forms part of a specific PSII protein complex (Campos et al., 2010).

It has been suggested that remodeling of grana may be possible through overexpression of a TGase and polyaminylation of antenna proteins, and this might play a functional role in the formation of the grana stacks and cause an imbalance between capture and use of light energy (Ioannidis et al., 2009; Ortigosa et al., 2010). A role for TGases in energy production in chloroplasts was recently suggested by analyzing the proton and electron circuit in thylakoids (Ioannidis et al., 2012), also showing that PU stimulates photophosphorylation while SD and SM are efficient stimulators of non-photochemical quenching (Ioannidis and Kotzabasis, 2007).

Polyamines were also found to be involved in the chloroplast development and dismantling. In fact, PAs exogenously added to *Z. mays* leaves during the transformation of etioplast to chloroplast accelerated the enzymatic conversion of protochlorophyllide to chlorophyllide, possibly stabilized through PAs, and the subsequent increased efficiency of photosynthesis (Beigbeder et al., 1995; Andreadakis and Kotzabasis, 1996). The mechanisms by which PAs can affect the assembly of plastidial membrane are partially mediated by their covalent linkage to these membranes via TGases (Sobieszczuk-Nowicka et al., 2007). The general drop in PA levels that occurred during the transformation of etioplast to chloroplast may be a result of the disappearance of a large prolamellar bodies to which PAs are bound for stabilization of its hexagonal structure.

Exogenous PA application or PA overexpression in plant cells also affect chloroplast metabolism during senescence and the forthcoming PCD. Even though it is difficult to compare the induced cell death with the natural one, experiments performed on the first system allow clarifying some events also occurring during natural senescence. Like SM, SD added to cut leaves of barley, senescing in darkness, inhibited the RNase activity, the degradation of chlorophyll and of LHCII protein (Legocka and Zajchert, 1999).

The fact that PAs are also effectively able to retard leaf senescence by their conjugation catalysed by TGase was directly shown in *Lactuca sativa* during induced cell death using leaf disks, or during the normal developmental senescence of leaves (Serafini-Fracassini et al., 2010). In leaf disks, supplied SM caused a delay of chlorophyll decay, an increase of endogenous TGase activity, and a threefold increase in chlorophyll content when supplied together with exogenous TGase. SM was conjugated, via TGase, mainly to 22–30 kDa proteins, a value shared by most of the antenna proteins. When the TGase activity was checked on *Lactuca* leaf left on the plant, it was higher in young leaves in respect to already-senescing ones. In young leaves, TGase was immunodetected in protein SDS gel bands of molecular mass of 77, 58, 39 and 20–24 kDa, close to those detected in chloroplasts of several other leaves (Serafini-Fracassini and Del Duca, 2008). In contrast, higher bands (160 kDa) were found in more senescent leaves (Serafini-Fracassini et al., 2010). A similar form was only detected in very low amount in chloroplast stroma fractions (Dondini et al., 2003).

When young and senescing *Lactuca* leaves, left on the mother plant, were SM-sprayed, an increase in free SM occurred suddenly in the young leaves, whereas over longer periods (15 days) there was an increase in perchloric acid-soluble and -insoluble SM metabolites. In already-senescing leaves, SM prevented degradation mainly of chlorophyll *b*, increased TGase activity and PA–protein conjugates, and maintained the leaf in a visible younger state. SM was conjugated mainly to LHCII by an endogenous TGase enzyme (co-fractionated with LHC) more significantly in the light, even though its conjugation occurred also in isolated PSI fractions (Serafini-Fracassini et al., 2010), in agreement with the 39 kDa TGase found to co-fractionate with maize LHCII (Della Mea et al., 2004a).

When [^3^H] SM was spotted as a tracer on leaf epidermis, its conjugation to the natural substrate of the leaf was observed: SM is transferred to chloroplasts and converted into the lower mass PAs, as SD and PU. When SM was sprayed on the young leaves, *mono*- and *bis*-PU and *bis*-SD were immediately produced more efficiently as compared to the control. On the contrary, if the sprayed leaves were left on the plant for additional 15 days and then extracted, only *bis*-PU was produced in higher amount. In SM-treated senescing plants, *mono*- and *bis*-PU and *bis*-SD and some additional derivatives, among which *bis*-SM, were produced in higher amount in respect either to the control or the SM-treated young leaves. Samples collected after 15 days are shown in **Figure 4C** (Serafini-Fracassini et al., 2010).

The protecting effect of SM on chlorophyll degradation could be related to its non-enzymatic binding (Dondini et al., 2003), either as free form but also as *mono*-PU derivative by its free primary amino group. As chlorophyll *b* is also linked to a glutamine of LHCII, this binding could further enhance the complex protein–chlorophyll stability and delay its degradation.

In summary, these data show that TGase activity, which is declining in untreated sample, is stimulated by SM to the level in young leaf; the effect is clearly visible with the endogenous chloroplast substrate and the senescing samples were also very reactive after late excision. The senescence-delaying effects of SM could be mediated by TGase protecting leaves from the decay of their chloroplast photosystem complexes.

In another leaf system, the excised barley leaf maintained in dark condition to cause its senescence, the level of PAs bound to thylakoids changed in senescing leaves: bound PU and SD increased throughout senescence, whereas bound SM decreased (Sobieszczuk-Nowicka et al., 2009). The decrease in bound SM during thylakoid degradation could be related to the breakdown of chloroplasts, degradation of LHCPII as well as other proteins of the chlorophyll *a/b* antenna complexes. An increase in TGase activity was detected by the colorimetric assay by using dimethylcasein as the substrate, an animal substrate frequently used also in plant assays to evaluate the enzyme levels. As the natural substrate is decreasing, the competition between the two substrates could justify the TGase increase. The immunodetection of TGase in thylakoid fraction revealed three bands of 33, 58, and 78 kDa whose intensity increased during senescence, showing a good correlation with the activity detected.

Kinetin supplied to petioles of excised barley leaves retarded senescence and diminished the increase in thylakoid-bound PU and SD and almost completely abolished the decrease of bound SM. These data suggest different roles of PU/SD and SM in thylakoid degradation. Kinetin down-regulated the accumulation of the 58- and 78-kDa TGases and the TGase activity but stimulated the presence of immunodetected thylakoid CP 26 used as a marker for the timing of thylakoid degradation. This could have an impact on the measure of TGase activity. The authors postulate that the formation of covalent bonds between PAs and proteins by TGase is involved in chloroplast senescence. The kinetin-mediated preservation of low TGase levels and activity throughout leaf senescence may represent an important component of the mechanism of kinetin action in the retardation of leaf senescence (Sobieszczuk-Nowicka et al., 2009).

### THE POLLEN PCD

The pollen tube is an excellent cell model to study the processes related to stress and cell death. The pollen tube is a cell destined to die as it expires after transporting the sperm cells to the embryo sac. Although this event is crucial to complete the process of fertilization, very little is known about it. Many more information are available on the self-incompatibility (SI) cell death, which is essential to prevent a plant to auto-fertilize thereby allowing plants to interbreed and therefore to increase genetic variability. During SI, the self-pollen is rejected after contact and, eventually, growth in the female tissues (stigma and style), while the non-self-pollen can survive and grow allowing fertilization. The SI process is precisely controlled at genetic level and is extremely selective (Rea and Nasrallah, 2008).

To perform its function, the pollen tube can grow through the stigma and style by “tip growth” (Cole and Fowler, 2006) a process by which secretory vesicles accumulate in the apical growing area and fuse with the plasma membrane releasing what is required for cell growth. A signal transduction system is necessary to allow pollen tubes to grow directionally toward the embryo sac while the cytoskeleton implements the information received from the transduction system and determines the accumulation of secretory vesicles (Šamaj et al., 2006). The oscillating changes of Ca^2^^+^ concentration at the apex are required to finely adjust the polymerization state of cytoskeletal elements (mainly actin) and consequently to control the flow of organelles and vesicles (Cole and Fowler, 2006). Any physical, chemical, or biological agent (such as SI) capable of altering this delicate mechanism can modify the pollen tube growth up to the dramatic consequence of blocking the process of fertilization.

Currently, little information is available on the relationship between cell death of pollen tubes and TGase/PAs. It is known that a form of extracellular TGase is involved in the apical growth of pollen tubes in apple tree. Since both specific inhibitors and monoclonal antibodies against TGase can block the growth of pollen tubes, TGase may play a role in the construction of the cell wall and in the interaction between pollen tubes and styles during fertilization (Di Sandro et al., 2010). In the pollen of *Malus domestica*, two polypeptides with a mass of 70 and 75 kDa were identified by immunoblotting with monoclonal antibodies against heterologous TGase. These proteins are able to cross-link both actin and tubulin thereby generating a number of products with a higher molecular mass (from 90 to 160 kDa). An additional 55 kDa immunoreactive polypeptide of the cell wall fraction has the same molecular mass as an active TGase extracted from the *Nicotiana* petal cell wall as reported above (Del Duca et al., 2009; Di Sandro et al., 2010).

Three main mechanisms of SI have been characterized in Angiosperms but only two of them operate at the level of pollen tubes, while the third mechanism works primarily at the level of non-germinated pollen grain. In poppy (*Papaver rhoeas*), the SI response requires a recognition event between specific S proteins of the stigma and pollen (Rudd and Franklin-Tong, 2003). In turn, this event triggers a cascade of Ca^2^^+^-dependent signals (many of which are unknown) that inhibit the apical growth of pollen tubes, producing critical changes in the trafficking of organelles and causing the depolymerization of actin filaments (with concomitant formation of actin foci; Thomas et al., 2003b). This process ends with the activation of a caspase-like protease activity. In the Solanaceae, Rosaceae, and Plantaginacee, the SI response is based on the presence of S-RNases, small proteins with RNAse activity that are produced by the pistil and are internalized in the pollen tube by either direct absorption or endocytosis (Wang et al., 2003). This system can be exemplified by the maloideae. In pear, the SI response is based on the internalization of specific proteins, called S-RNase (Liu et al., 2007), through hypothetical endocytotic processes. In incompatible pollen tubes, S-RNases would be released from vacuoles and free up in the cytoplasm where they degrade mRNA (Goldraij et al., 2012). The growth of incompatible pollen tubes would not be blocked exclusively by the degradation of mRNA, as other degradative processes will take part. In pear, the SI response also affects the activity of mitochondria, leading to changes in the production of ROS (reactive oxygen species) and therefore to alterations of the growth process (Wang et al., 2010). In turn, ROS (by affecting the levels of Ca^2^^+^) may cause substantial changes to actin filaments (Liu et al., 2007; Wang and Zhang, 2011). Thus, blocking the growth of incompatible pollen tubes requires mechanisms that are partly similar to the SI response of poppy. In the case of pear, the disorganization of actin filaments would lead to the breakdown of vacuoles and to the release of S-RNase in the cytoplasm. Although no information is available on the role of microtubules in the SI response in pear, recent data obtained in apple indicate that microtubules could be critical in the internalization process of S-RNase by driving endocytotic membranes toward the vacuole system in order to release S-RNase in the pollen tube cytoplasm (Meng et al., 2014).

**FIGURE 5:**
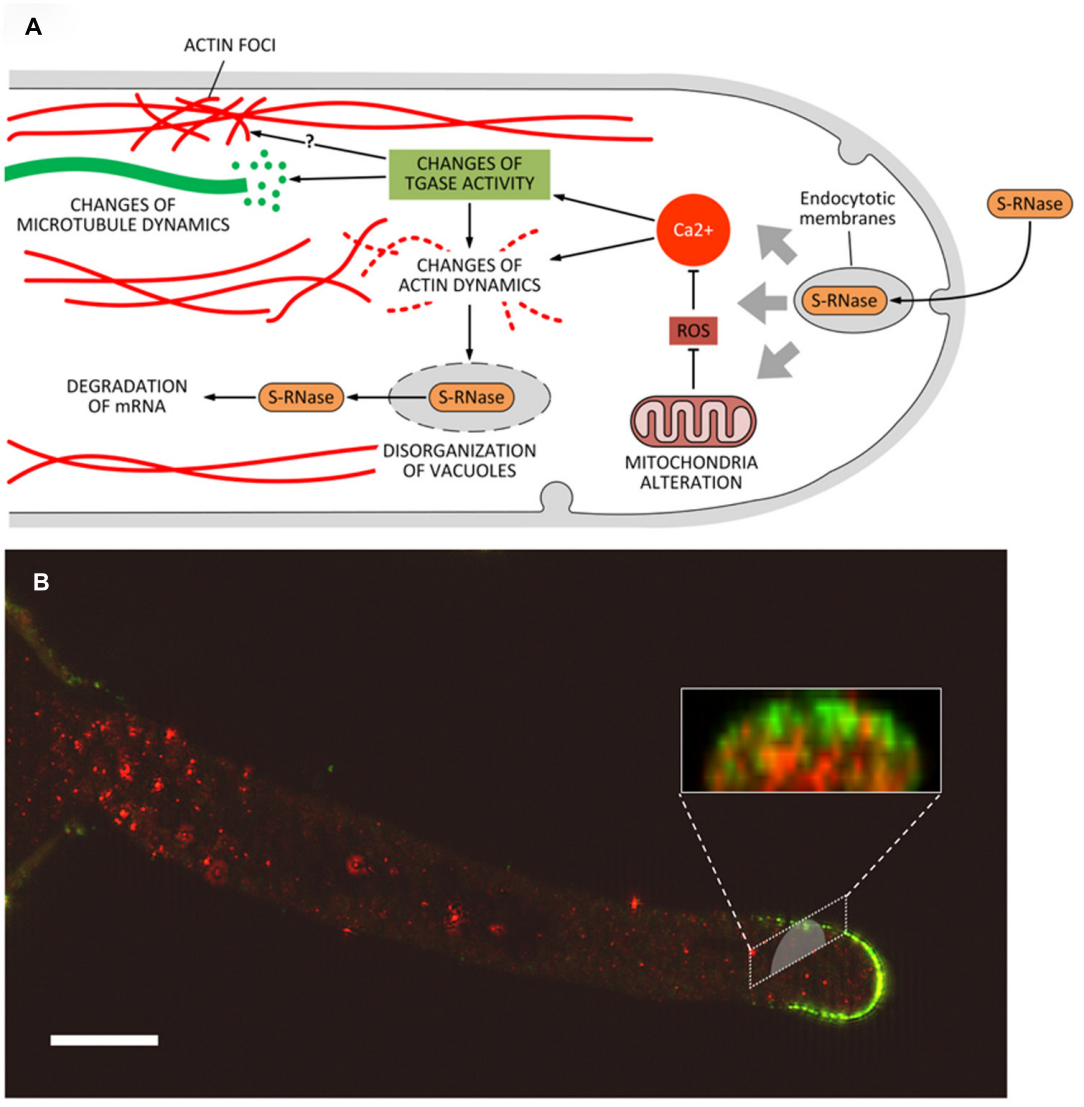
**(A)** Model of the potential role of transglutaminase (and PAs) during the process of self-incompatibility in pear. S-RNase would be incorporated by endocytosis. This process would trigger a series of subsequent events, including the alteration of mitochondria morphology and consequently the production of ROS. In turn, abnormal levels of ROS might modify the intracellular concentration of Ca^2^^+^ thus leading to substantial modifications in the structure of actin filaments and/or to interference in the activation/regulation of TGase. As a result, further changes at level of actin (formation of actin foci) and microtubules may also occur. Along with the degradation of mRNA caused by S-RNase released in the cytoplasm, these effects contribute to the death of pollen tubes. **(B)** Distribution of TGase in pollen tubes and relationship with cell wall components. In pollen tubes, TGase (in red) is found in the cytoplasm but also in the cell wall where the enzyme could increase the stiffness of cell wall, contributing to counteract the internal turgor pressure. A further role of TGase during the SI response could be related to the interaction between TGase and specific cell wall components (such as arabinogalactans, in green) as part of the SI response. This interaction may thus prevent the growth of incompatible pollen tubes. Bar: 10 μm. The method for immunolabeling and similar figures can be found in Del Duca et al. (2013a).

What is the role of PAs and TGase in the above-mentioned processes? TGase has not been characterized in the pollen tube of poppy; therefore, the following assumptions mainly relate to the pollen tube of pear where TGase has been identified and characterized from different points of view (Del Duca et al., 2013a). Data on the possible relationship between TGase and incompatibility were also obtained in *Citrus* (Gentile et al., 2012). In *Citrus*, an increase in the content of either bound or conjugated PAs and of TGase activity occurs during incompatible pollination reaching a peak when the process of SI becomes visible (**Figure 4C**; Gentile et al., 2012). Immediately after, TGase activity decreases. In contrast, during compatible pollination the TGase activity decreases rapidly and then stabilizes approximately on the values of incompatible pollination. In pear, all the TCA-insoluble PAs increase after fertilization while SM and PU are higher in incompatible pollination (Del Duca et al., 2010). These data would be in agreement with a possible increase of TGase as observed in *Citrus*. The increase of TGase activity in the incompatible pollination of pear does not seem dependent on a higher expression of the enzyme (Iorio et al., 2012a) but it is probably related to changes in the concentration of intracellular Ca^2^^+^ occurring during the SI response (Wang et al., 2010). Since TGase is a Ca^2^^+^-dependent enzyme, changes in the concentration of Ca^2^^+^ as induced by the SI response may significantly alter the enzymatic activity of TGase. A further support for the role of TGase during the SI response comes from the discovery that cytoplasmic TGase of apple pollen is able to post-translationally modify actin and tubulin by conjugating with PAs (Del Duca et al., 1997). Such activity would result in the generation of high-molecular-weight aggregates (Del Duca et al., 2009) capable of altering the dynamic properties of the cytoskeletal filaments, of reducing the affinity of kinesin and myosin and, consequently, of affecting the dynamic activities based on the two motor proteins. Thus, TGase might actively participate in the SI response by playing a critical role in the reorganization of the cytoskeleton (**Figure 5A**). Changes in the Ca^2^^+^ concentration after the onset of SI response can also modify the functional properties of actin filaments and microtubules (Liu et al., 2007) through the enzymatic activity of TGase. Therefore, the molecular mechanisms of rejection of SI pollen may share common features among different families, such as poppy and pear.

An additional role for TGase during the SI response may also be found in the interaction between TGase and the cell wall. In incompatible pollen tubes of pear, TGase forms a sort of “cap” around the apex of incompatible pollen tubes (Del Duca et al., 2010); as it occurs also in *Citrus*, these data may suggest that TGase increases the rigidity of the apical cell wall, thus counteracting the internal turgor pressure and preventing pollen tubes to grow further. In addition, TGase was also observed to accumulate occasionally as consistent aggregates in the cell wall of pollen tubes (Iorio et al., 2008; Di Sandro et al., 2010; Del Duca et al., 2013a; Del Duca et al., 2014). These aggregates could hypothetically be involved in different processes, such as the modification of specific glycoproteins and polysaccharides of the cell wall (**Figure 5**). Experiments of double immunofluorescence showed co-localization between TGase and other specific cell wall components, including arabinogalactans and pectins (**Figure 5B**; Del Duca et al., 2013a). The mechanism by which TGase is secreted into the cell wall is not known and may require non-canonical mechanisms of secretion, as discussed by Del Duca et al. (2013b). Whatever the process of secretion, TGase could actively participate in changes of the cell wall during the SI response thereby leading to growth arrest of incompatible pollen tubes. However, we cannot exclude that these aggregates are simply the result of an altered secretion process of extracellular TGase due to the SI response.

### THE HYPERSENSITIVE RESPONSE

In another type of PCD, during the hypersensitive response induced by tobacco mosaic virus, (TMV) free and conjugated PAs increased their concentration together with their biosynthetic enzymes (Torrigiani et al., 1997). At difference with the mock-inoculated samples, *mono*-PU and *bis*-SD were recovered after TMV-inoculation, which further on increased. A putative 72 kDa-TGase immuno-recognized by AtPng1p polyclonal antibody, increased in TMV-inoculated leaves and in the lesion-enriched areas. TGase activity was found to increase in the intrinsic membrane protein and in cell wall fractions, and it was more persistent in TMV-inoculated leaves (Del Duca et al., 2007). A possible role in defense by TGase against virus by isolating the infected areas was proposed in agreement with data in mammalian cells where a number of interacting viral and cellular proteins have been found to be modified by TG2, suggesting its novel function in viral pathogenesis (Jeon and Kim, 2006). This role of TG to isolate safe cells from possible damaging organisms recall the isolation of cells from dying ones by isolating their fragments, thus preventing leakage of macromolecules before clearance in the apoptotic bodies (Fesus and Piacentini, 2002).

### THE TUBER SENESCENCE AND DEATH

**FIGURE 6:**
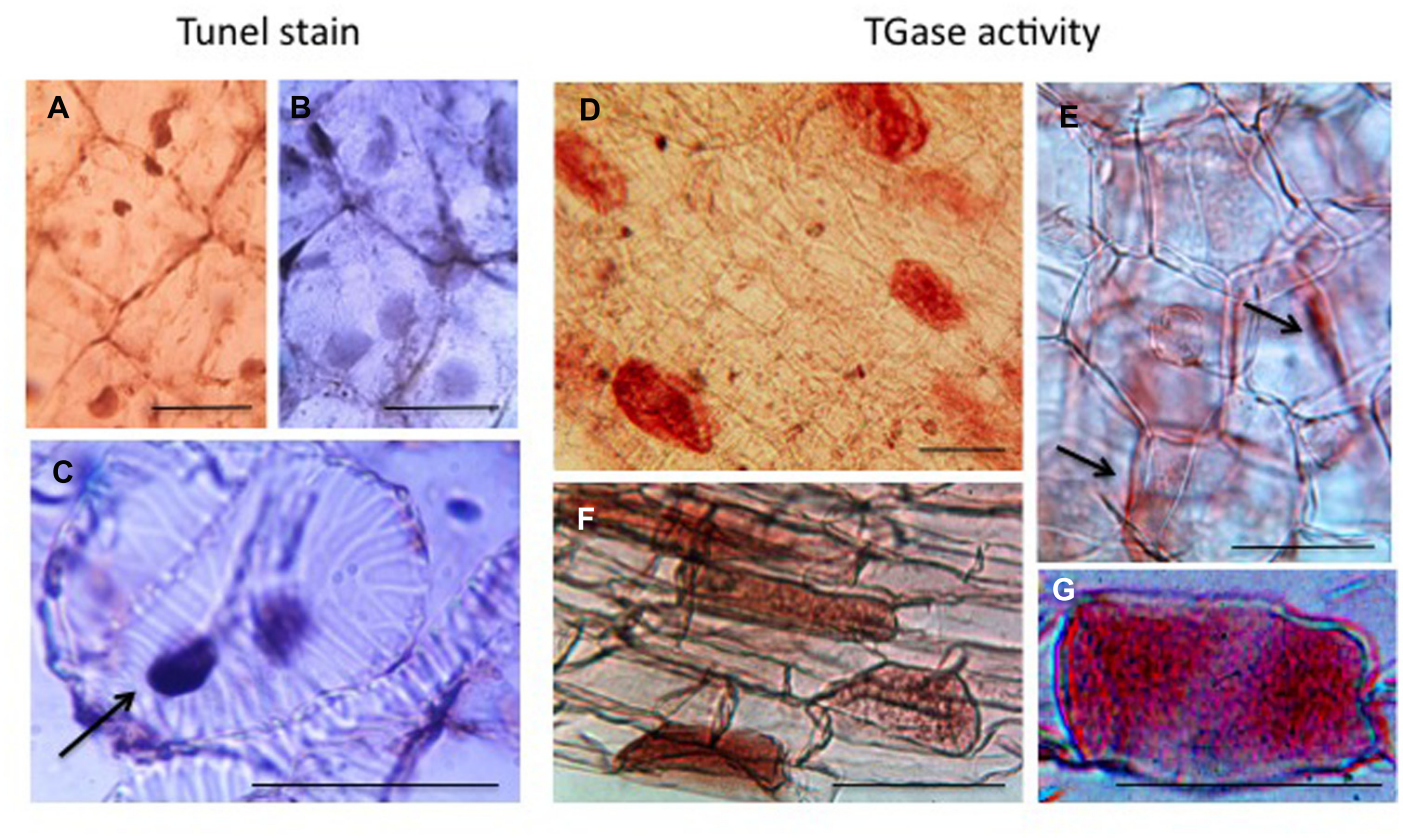
**Programmed cell death (PCD) and transglutaminase activity in the storage parenchyma of the tuber of *Helianthus tuberosus* during dormancy release.** Scattered cells either parenchymatic **(A)** or tracheary elements undergoing PCD (**C**, arrow) were positive to Tunel staining. Other cells were negative **(B)**. Fresh tuber slices were also incubated with dansyl-cadaverine and after extensive washing, immuno cytochemically recognized by an anti-dansyl antibody. Cells positive to conjugated cadaverine were scattered among others (**D** and **F**). TGase products were found either mainly in the cell walls (**E**, arrows) or in the whole cells (**G**). Bars: 50 μm.

The last example of PCD, probably better defined as DCD, refers to the tubers that at the time of dormancy release become depleted of their storage substances and die when the translocation of nutrients to growing roots and sprouts is completed. In *Helianthus tuberosus*, this organ provides a homogeneous tissue: the medullary parenchyma that stores different compounds like several aminic and glucosidic substances. Cells are characterized by a thin layer of cytoplasm, which includes nuclei, adherent to the thin cell wall, with small nucleoli and a large vacuole. Their non-green plastids are small and contain tubular complexes (**Figure 6**). Even though the winter dormancy of the tuber is not an absolutely stationary period, the large parenchyma cells (arrested in G0 phase) have a slow metabolism, contain very small amounts of growth substances, but a considerable amount of inhibitors. PAs are also present but in insufficient amount to sustain growth and TGase activity is low. For this reason, the dormant parenchyma represents a natural PA-deficient tissue and was utilized to test *in vitro* for the first time the effect of supplied PAs on plant growth (Bertossi et al., 1965). Explants, when put in *in vitro* culture, immediately respond activating many metabolisms, included the biosynthetic and catabolic ones of PAs, their linkage to RNAs and their conjugation by TGase activity especially to very high molecular mass conjugates and to a 18-kDa protein. New TGase enzymes are synthesized either of 58 and 90 kDa but also of higher mass (Serafini-Fracassini et al., 1988; Del Duca et al., 2000). Cells activate the metabolisms of DNA and of different RNAs, and enter the first synchronous amitotic cell cycle continuing to grow, as partially summarized by Bagni and Serafini-Fracassini (1985) and by Tassoni et al. (2010). However, this treatment caused the break of dormancy, which consists of an induced change of the death program of parenchyma cells, many of which become meristematic. On the contrary, during the natural release from dormancy, parenchyma cells are programmed to become totally depleted and die: in fact storage substances decrease. This phase is gradual and long, the system is rather complex involving the development of sprouts and roots and the transfer of nutrients and growth factors therein (Grandi et al., 1992). Unexpectedly, the tuber cells die randomly. In fact, when observed after Tunel staining, parenchyma cells and differentiating tracheids with dark picnotic nuclei are interspersed with cells with normal nuclei (**Figures 6A–C**). Tracheid differentiation could favor the transfer of storage substances.

The amount of bound PU and SM increases during dormancy up to dormancy release and decreases during sprouting in conjunction with a sudden rise of free PAs. Most of the PAs were linked to proteins and possibly are transferred to growing sprouts (Serafini-Fracassini and Mossetti, 1985; Mossetti et al., 1987). At the time of sprouting, a conspicuous increase in protein content of tubers takes place followed by a slow decrease. A 47-kDa protein band increased at the end of dormancy and then decreased during sprouting. Among the high molecular mass bands, a 150-kDa band increased dramatically at dormancy release. Numerous additional bands in the 35–50 kDa range appeared only in this phase (Del Duca et al., 2000). These changes could be related either to the synthesis of degradative enzymes and to released storage substances to be transferred to growing sprouts or to the PCD of tuber parenchyma, which has completed its function.

TGase activity, as measured in tuber cell-free system, increased during dormancy and dropped at sprouting (Grandi et al., 1992). During this phase, this activity was localized scattered only in few parenchyma cells as shown by immuno-cytochemical recognition of conjugated dansyl-cadaverine (**Figure 6D**). This substrate appeared distributed in the whole cell (**Figures 6D–G**) but also in the cell walls (**Figure 6E**). The distribution of these labeled cells recalls the distribution of Tunel positive cells. It can be hypothesized that the same tuber cells, which die randomly, are modified by TGase similarly to the hepatocytes or apoptotic bodies, which are isolated by a net of proteins conjugated by TG2 to protect the surrounding cell from a release of dangerous enzymes (Fesus and Piacentini, 2002).

This example of PCD clarify that it is necessary to use an homogeneous plant material and to verify PCD distribution by morphological methods, otherwise the biochemical data, that provide a mean value of cells in different metabolic stages, might be misleading. In fact, PAs conjugated by TGase are also present in this PCD system, suggesting that this is a widespread event in PCD.

## CONCLUSION

Among the plant models described here, some may be attributed to a type of PCD classified as “vacuolar cell death”, others to the so-called “necrotic cell death” or, finally, to a mixture of both according to the classification proposed by van Doorn et al. (2011). During vacuolar cell death, the cell contents are removed by a combination of autophagy-like process and release of hydrolases from collapsed lytic vacuoles. Necrosis is characterized by early rupture of the plasma membrane, shrinkage of the protoplast and absence of vacuolar cell death features. Vacuolar cell death is common during tissue and organ formation and elimination, whereas necrosis is typically found under abiotic stress. However, this classification is based only on morphological observations, not always available; the proper diagnose of vacuolar cell death should be obtained by combining electron microscopy observations together with analysis of autophagic activity of the vacuolar processing enzymes and with changes of the cytoskeleton. Morphological events occurring during vacuolar cell death include the assembly of actin bundles, the breakdown of the nuclear envelope, and even nuclear segmentation. According to van Doorn et al. (2011), vacuolar cell death was previously observed in tracheary elements or in pollen as well as in petals (where we also observed events of DNA laddering and nuclei fragmentation); consequently, vacuolar cell death might occur also in the models here presented. All the characteristics of vacuolar cell death and necrosis can be tracked during the HR cell death; in addition, a mixture of necrotic and vacuolar characteristics also occurs during the SI response, possibly even in the models presented here. Necrosis, no longer considered an unprogrammed process, remains poorly characterized at the biochemical and genetic levels and, although there are no molecular markers, it can be defined as PCD.

The evidence for different TGase forms, some of which are specific for a particular organelle or structure, implies that there must be also different substrates. A role of TGase more frequently observed is linked to the stabilization of structural proteins that, when modified by TGase, are more protected from digestion by proteases. As an example, a cytoplasmic TGase modifies cytoskeletal proteins in pollen, and changes to the cytoskeleton are typical features of PCD.

The effect of TGase is probably related to the type of plant PCD but mostly to the substrate to be modified in order to achieve that specific PCD program. TGase activity increases during natural senescence of the papyraceous petals of *Nicotiana*, whose cell walls become probably lignified or suberified, and during SI pollination, when the pollen apex is covered by a thick cap, or during the PCD induced by hypersensitive response against tobacco mosaic virus, where a defense suberified layer is formed. Perhaps a similar defense role but against the degradative enzymes occurs also in tuber cells. In contrast, TGase activity appears to decrease during natural (developmental) senescence of not-excised old leaves. This difference may depend on changes occurring mostly in the cell wall; in fact, the latter is often involved in PCD, as in tracheids. In contrast, in senescent leaves this modification probably does not occur. Moreover, from our experience, it is critical the age and thus the metabolic stage of the senescent organ examined. In photosynthetic tissues, the stabilizing role of TGase occurs mainly on the proteins of chloroplasts; this event is essential to sustain the photosynthetic activity in order to make the energy available for cell metabolism, also that of senescing leaves or petals, whereas in more senescent ones energy is no more necessary and thus TGase activity declines. In not-excised senescing leaves, senescence is reversible as the addition of PAs stimulated TGase activity and maintained this organ in a state of juvenility. Also in the two models of leaf PCD induced by excision, TGase changed but it could be conditioned by many “accidental” experimental events, like wounding, water availability, dark or light, partial anoxia, effects of different supplied factors (PAs, kinetin, exogenous TGase). Therefore, it is difficult to obtain a clear unambiguous picture.

In all models of plant PCD hitherto examined, TGase appears to be involved in a way to some extent similar to some of those described during apoptosis in animal cells, in particular when TGase catalyzes the posttranslational modification of proteins by transamidation, with consequent formation of cross-links and even large supramolecular nets in which PAs may be involved. However, the role of TG2 in the animal cell death/survival process is extremely complex, and circumstances in which TG2 acts as a prodeath or prosurvival protein is still an area of active investigation (Gundemir et al., 2012). Thus, scarce suggestions can be obtained by animal cell models. Moreover some typical characteristics of animal “apoptosis”, a term not used in plants, cannot be detected in plants due to differences at cellular, tissue, and organismic levels, although many properties are shared between animals and plants at molecular level.

Much more information is required to get an overall picture of the role of PAs and TGase during PCD in plant cells. However, it is clear that TGase is involved in all models presented here and that, as a cross-linker of PAs and proteins, it is an important factor involved in multiple, sometimes controversial, roles of PAs during PCD.

## Conflict of Interest Statement

The authors declare that the research was conducted in the absence of any commercial or financial relationships that could be construed as a potential conflict of interest.

## ABBREVIATIONS

AZ: abscission zone
ADF: actin-depolymerizing factors
*bis*-PAs: *bis*-(γ-glutamyl)-PAs
CAP: cyclase-associated proteins
DCD: developmental cell death
HR: hypersensitive response
LHCII: light-harvesting complex II
LHC-P: light-harvesting-proteins
*mono*-PAs: *mono*-(γ-glutamyl)-PAs
PAs: polyamines
PCD: programmed cell death
PU: putrescine
ROS: reactive oxygen species
RuBisCO: ribulose-1,5-bisphosphate carboxylase/oxygenase
SI: self-incompatibility
SD: spermidine
SM: spermine
S-RNase: self-RNase
tTGase or TG2: tissue transglutaminase
TMV: tobacco mosaic virus
TGase: transglutaminase.
